# Preliminary outcomes of boron neutron capture therapy for head and neck cancers as a treatment covered by public health insurance system in Japan: Real‐world experiences over a 2‐year period

**DOI:** 10.1002/cam4.7250

**Published:** 2024-06-03

**Authors:** Satoshi Takeno, Yuki Yoshino, Teruhito Aihara, Masaaki Higashino, Yasukazu Kanai, Naonori Hu, Ryo Kakino, Ryo Kawata, Keiji Nihei, Koji Ono

**Affiliations:** ^1^ Department of Radiation Oncology Osaka Medical and Pharmaceutical University Osaka Japan; ^2^ Kansai BNCT Medical Center Osaka Medical and Pharmaceutical University Osaka Japan; ^3^ Department of Otorhinolaryngology – Head and Neck Surgery Osaka Medical and Pharmaceutical University Osaka Japan; ^4^ BNCT Joint Clinical Institute Osaka Medical and Pharmaceutical University Osaka Japan; ^5^ Institute for Integrated Radiation and Nuclear Science Kyoto University Osaka Japan

**Keywords:** boron neutron capture therapy, boron, head and neck cancer, re‐irradiation, radiation therapy

## Abstract

**Purpose:**

Since June 2020, boron neutron capture therapy (BNCT) has been a health care service covered by health insurance in Japan to treat locally advanced or recurrent unresectable head and neck cancers. Therefore, we aimed to assess the clinical outcomes of BNCT as a health insurance treatment and explore its role among the standard treatment modalities for head and neck cancers.

**Materials and Methods:**

We retrospectively analyzed data from patients who were treated using BNCT at Kansai BNCT Medical Center, Osaka Medical and Pharmaceutical University, between June 2020 and May 2022. We assessed objective response rates based on the Response Evaluation Criteria in Solid Tumors version 1.1, and adverse events based on the Common Terminology Criteria for Adverse Events, version 5.0. Additionally, we conducted a survival analysis and explored the factors that contributed to the treatment results.

**Results:**

Sixty‐nine patients (72 treatments) were included in the study, with a median observation period of 15 months. The objective response rate was 80.5%, and the 1‐year locoregional control, progression‐free survival, and overall survival rates were 57.1% (95% confidence interval [CI]: 43.9%–68.3%), 42.2% (95% CI: 30.1%–53.8%), and 75.4% (95% CI: 62.5%–84.5%), respectively. Locoregional control was significantly longer in patients with earlier TNM staging and no history of chemotherapy.

**Conclusions:**

BNCT may be an effective treatment option for locally advanced or recurrent unresectable head and neck cancers with no other definitive therapies. If definitive surgery or radiation therapy are not feasible, BNCT should be considered at early disease stages.

## 1 INTRODUCTION

Boron neutron capture therapy (BNCT) is a treatment in which compounds containing boron (^10^B) are incorporated into tumors followed by subsequent irradiation with thermal neutrons.^1^ This causes a boron neutron capture reaction (^10^B(n, α)^7^Li) within the cells that have incorporated the boron compounds, resulting in high‐linear energy transfer (LET) particles that destroy the tumor. These high‐LET particles have a range of <10 μm, which is shorter than the diameter of a single cell. Therefore, they can selectively destroy tumor cells while minimizing damage to neighboring cells, even if the tumor is invasive and actively spreading.^2^, ^3^

Considering these mechanisms, the boron compound is key to demonstrating the therapeutic efficacy of BNCT. Although new boron compounds are being developed,^4^ in clinical practice, 4‐borono‐L‐phenylalanine (BPA) is widely used as a boron compound.^5^ It is known to be selectively taken up by tumors via the L‐type amino acid transporter 1 (LAT1),^6^ which is upregulated in many cancers.^7^

Dose evaluation is essential in any radiation therapy planning, including BNCT. The main dose component in BNCT is the boron neutron dose, which is the doses from the high‐LET particles generated by boron neutron capture reaction. It is evaluated by converting into an x‐ray equivalent dose (Gy‐Eq) based on the neutron dose, boron concentration, and tissue sensitivity. The neutron dose is calculated using Monte Carlo simulations. The boron concentration in normal tissues is assumed to be equal to the blood boron concentration. Additionally, the tumor boron concentration is converted from the blood boron concentration using the ratio of the tumor boron concentration to blood boron concentration (tumor/blood [T/B] ratio). Tissue sensitivity to boron neutron doses in BNCT is commonly assessed using the compound biological effectiveness (CBE) factor—a concept similar to relative biological effectiveness (RBE).^8^

BNCT for head and neck cancers began in Japan in 2001 ahead of the rest of the world,^9^ and thereafter, has been used in Finland and Taiwan.^10^, ^11^ Previous trials have shown satisfactory results^12^, ^13^; however, at the time, neutron irradiation required a nuclear reactor, therefore BNCT could not be used as a general treatment and remained merely a special‐case experimental procedure. To address this clinical difficulty, an accelerator (cyclotron)‐based BNCT system was developed by the Kyoto University Group in collaboration with Sumitomo Heavy Industry, Ltd. (Tokyo, Japan) in 2008.^14^ Following the JHN002 study which showed that accelerator‐based BNCT for locally advanced or recurrent unresectable head and neck cancers had a favorable response rate of 71%,^15^ the Ministry of Health, Labour, and Welfare in Japan approved accelerator‐based BNCT as a treatment covered by general health insurance. Thus, Japan was the first country to implement BNCT through its health insurance system in June 2020.

To the best of our knowledge, this study is the first report on the safety and clinical effectiveness of BNCT in Japan, following its coverage by the country's health insurance system. Although there are a number of treatment options for head and neck cancers—including surgery, radiation therapy, and chemotherapy—their results remain underwhelming. As BNCT is now a viable treatment option in Japan, we aimed to evaluate the contribution of BNCT to standard treatment, particularly for locally advanced or recurrent unresectable head and neck cancers, based on our clinical experiences.

## 2 MATERIALS AND METHODS

### 2.1 Determination of treatment planning parameters for BNCT

When calculating BNCT doses, information on T/B ratios and CBE factors is required, in addition to the neutron dose. However, there is no currently available method to measure the T/B ratio in standard clinical practice. It is generally assumed that a T/B ratio of ≥2.5 is recommended for the treatment of BNCT.^16^ Moreover, a previous study also revealed the T/B ratio was ≥2.5 for majority of the tumors.^17^ Therefore, we set the T/B ratio to 2.5. In terms of CBE factors, the values for the tumor, pharyngeal mucosa, skin, and other normal tissues were set to 3.8, 4.9, 2.5, and 1.35, respectively, based on previous studies.^18^, ^19^, ^20^, ^21^ As there is currently no consensus on the value of the T/B ratio and the tumor CBE factor, different values are used in this study than in the JHN002 trial. This point is discussed in detail in the Discussion section.

Although the T/B ratio and CBE factors differ for every tumor, they were calculated using the fixed values listed above, because currently there is no method to individualize them in daily practice. This implies that uncertainty regarding tumor dose is inevitable. However, normal tissue is generally considered to be homogeneous; thus, the fixed T/B ratio and CBE factor values selected were assumed to be reliable. Therefore, the prescribed dose for each patient was determined based on the normal tissue dose, to maximize the dose within the normal tissue‐tolerable range. The tolerable normal tissue doses were set to 15, 12, 9, and 5 Gy‐Eq for the skin, pharyngeal mucosa, brain, and eyes, respectively.

### 2.2 Patient selection and preparation for BNCT

We analyzed data from patients with head and neck cancers who were treated at the Kansai BNCT Medical Center, Osaka Medical and Pharmaceutical University (Osaka, Japan) between June 2020 and May 2022. Patients eligible for insured BNCT treatment in Japan included those with “locally advanced or recurrent unresectable head and neck cancers”—particularly those with no malignant lesions other than the target lesion. Here, the term “unresectable” includes surgical refusal, such as a desire to preserve the larynx. Further, we limited eligible patients to those who were expected to be curable based on pretreatment plans made prior to treatment preparations. In this study, curative dose was defined as an x‐ray equivalent dose of 20 Gy‐Equation (T/B ratio 2.5, CBE factor 3.8) delivered to at least 80% of the tumor volume.^22^ Therefore, we defined the eligible criteria as the equivalent dose of more than 20 Gy‐Equation (T/B ratio 2.5, CBE factor 3.8) delivered to the tumor volume of at least 80% in pretreatment plan.

If the tumor with skin or mucosal invasion is located adjacent to the carotid artery, the risk of carotid artery rupture is considered high; therefore, we excluded such cases from this study.^23^ Instead of BNCT, we recommended other effective standard treatments (such as surgery or radiation therapy) if they were available. Additionally, patients with a high risk of laryngeal edema based on previous treatment history and/or laryngeal findings underwent tracheotomy prior to BNCT.

This study was approved by the ethics committee of Osaka Medical and Pharmaceutical University (Study No. 2021‐172). Written informed consent for publication was obtained from each patient.

### 2.3 Treatment planning

For eligible patients, the patient setup positions were considered during the pretreatment planning, and computed tomography (CT) simulation was performed accordingly. Delineation of the target and organs at risk and the generation of the treatment plan for BNCT were created using RayStation® version 9A (Raysearch Laboratories AB, Stockholm, Sweden). Dose calculation was performed using NeuCure Dose Engine® (Sumitomo Heavy Industries).^24^

### 2.4 Boron administration and irradiation

Borofalan, a BPA‐based drug, was used as the boron compound because it was the only drug approved by the Japanese regulatory agency. Borofalan was administered according to Ono's method^25^: at a rate of 200 mg/kg/h for the first 2 h before irradiation, followed by a reduction to 100 mg/kg/h once the irradiation began, to maintain the boron concentration during treatment.

The NeuCure BNCT System® (Sumitomo Heavy Industries) was used as the neutron source.^26^ At the time of irradiation, the required BNCT dose is already determined, and the CBE factor is a fixed value. Therefore, if the boron concentration is determined, the neutron dose, that is, the irradiation time, can be determined. The blood boron concentration immediately prior to irradiation was measured via inductively coupled plasma atomic emission spectrometry (ICP‐AES) in order to determine the irradiation time. As the boron concentration can change during irradiation, the actual boron concentration was estimated as the average of the values immediately before and after irradiation, and a post‐plan was created based on this value to evaluate the actual delivered dose.

### 2.5 Follow‐up and assessment of treatment result

Posttreatment follow‐up was performed at our institution whenever possible; however, if the patient lived too far away, it was performed at the referring institution. The effectiveness of the treatment was evaluated as the best overall response after BNCT, based on the Response Evaluation Criteria in Solid Tumors v1.1, via CT, magnetic resonance imaging, and/or fluorodeoxyglucose positron emission tomography (FDG‐PET). Clinical findings were also obtained if available. Thereafter, follow‐up continued every 3 months whenever possible.

Grade 3 or higher adverse events were evaluated according to the Common Terminology Criteria for Adverse Events 5.0.

### 2.6 Statistical analysis

Locoregional control, progression‐free survival, and overall survival were analyzed using the Kaplan–Meier method. The duration of locoregional control was defined as the period from the date of irradiation to the date of disease progression at the irradiated site, and the duration of progression‐free survival was defined as the period from the date of irradiation to the date of disease progression or death. For patients who received BNCT twice, only the initial administration was included in this analysis.

The log‐rank test was then performed for T (T1 + T2 vs. T3 + T4) and N staging (N1 + N2 vs. N3) at the time of irradiation according to the Union for International Cancer Control TNM classification 8th Edition. In this analysis, patients with both primary and lymph node lesions were excluded, to focus separately on the effects of either T or N factors. Furthermore, log‐rank tests by histological type (squamous cell carcinoma [SqCC] vs. non‐squamous cell carcinoma [non‐SqCC]) were performed. A chi‐squared test was performed to assess the differences in TNM staging, as a potential confounding factor, in each group. Then, log‐rank tests were performed for pretreatment data focusing on SqCC cases, to evaluate the value of BNCT as a standard treatment for head and neck cancers. We also performed a chi‐squared test to assess the differences in TNM staging in this analysis. The reason for focusing on SqCC was to avoid confounding factors due to differences in histological type, as they can affect treatment efficacy. Statistical significance was set at *p* < 0.05. All statistical analyses were performed using Python 3.10, with a lifelines 0.27 library (Davidson‐Pilon, et al.).^27^

## 3 RESULTS

### 3.1 Patient and irradiation characteristics

Between June 2020 and May 2022, 69 patients underwent BNCT for head and neck cancer. Three of them underwent second BNCT for recurrence of the irradiated site after the first BNCT; therefore, a total of 72 treatments were performed. However, two of the treatments could not be completed because the patients were unable to maintain the irradiation position due to pain. One of these was resumed on a different day, thus, the final number of irradiation sessions was 73. The treatment planning procedure for the second BNCT was the same as the first. All 72 treatments were included in this analysis, even though some cases were not completed, as they were considered to have been irradiated to the curative dose. The irradiated tumor sizes ranged from 0.23 to 235.11 cm^3^, with a median size of 6.90 cm^3^.

**FIGURE 1:**
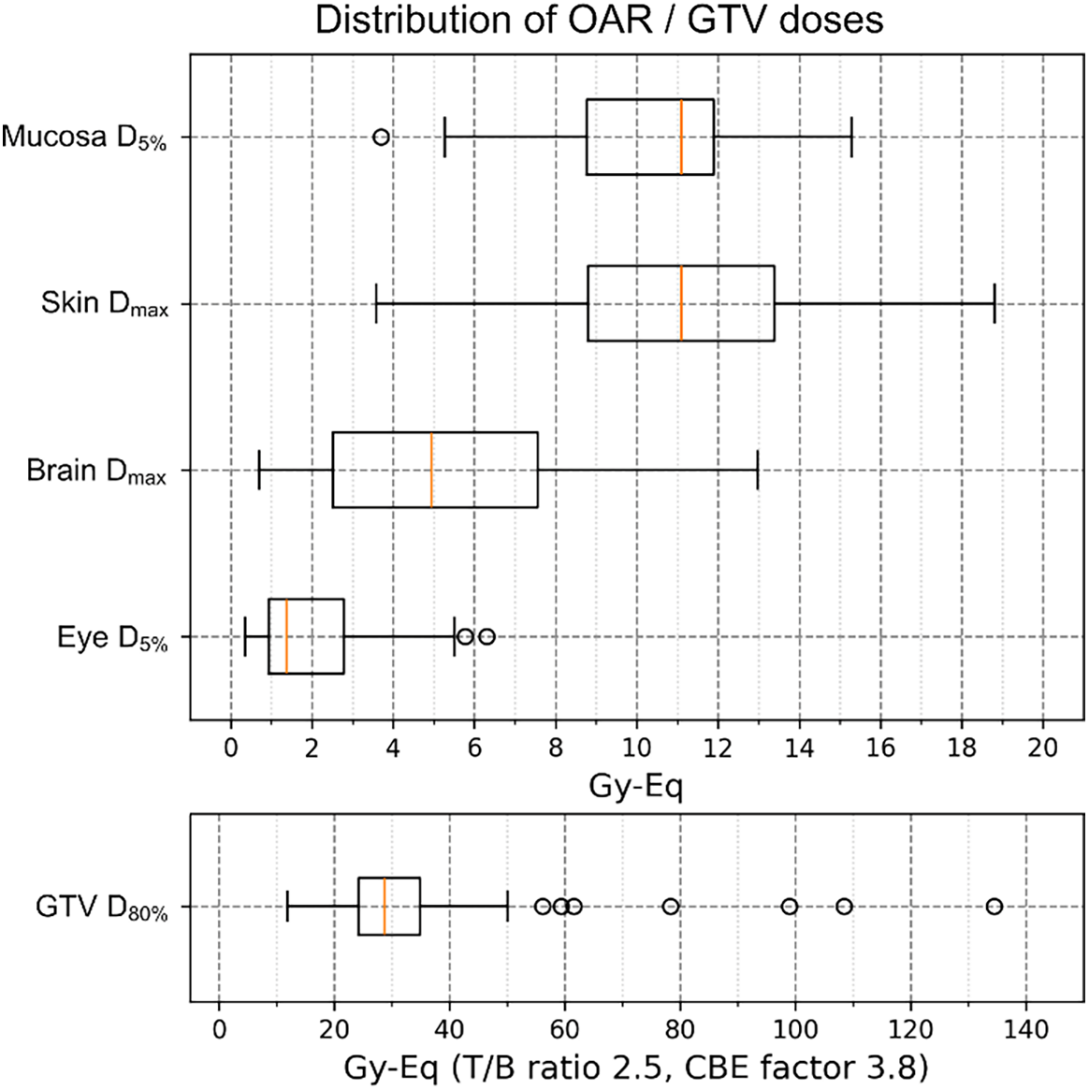
Distribution of each organ at risk (OAR) and tumor (GTV) doses for the 72 cases. OAR doses were evaluated at pharyngeal mucosa *D*_5%_, skin *D*_max_, brain *D*_max_, and eye *D*_5%_. GTV dose was evaluated at *D*_80%_. The box in the boxplot represents the interquartile range and the orange line represents the median.

Patient background data are presented in Table 1. The median age was 71 years, and male‐to‐female ratio was 43:26. The observation period for this analysis ranged from 0 to 37 months (median, 15 months). Table 2 shows the primary disease and histology characteristics of the 69 included patients, as well as their irradiation sites at the time of initial BNCT. Of the total patients who underwent initial BNCT, 4 were considered fresh treatments and the remaining 65 were considered recurrent. The distribution of risk organ doses and tumor doses and for the 72 cases analyzed in this study are shown in Figure 1.

**TABLE 1:** Patients' details.

Patient characteristics	
Median age (years) [range]	71 [40–89]
Male/female	43/26
Previous treatment	
Surgery	35 (50.7%)
Radiation therapy	58 (84.1%)
Chemotherapy	50 (72.5%)
Immunotherapy	23 (33.3%)
Median observation period (months) [range]	15 [0–37]

**TABLE 2:** Disease details.

Cancer	Cases	TN staging at irradiation									Pathology	
		T0	T1	T2	T3	T4	N0	N1	N2	N3	SqCC	Non‐SqCC^a^
Oral cancer												
Tongue	9	7	0	0	2	0	2	0	1	6	9	0
Others	11	0	0	2	0	9	10	0	1	0	10	1
Nasopharynx	5	1	1	3	0	0	4	0	0	1	5	0
Oropharynx	7	3	2	1	1	0	4	0	1	2	7	0
Hypopharynx	5	1	1	1	2	0	4	0	1	0	5	0
Laryngeal cancer	6	2	2	1	0	1	4	0	1	1	6	0
Nasal/paranasal cancer	6	0	1	1	0	4	5	1	0	0	5	1
Salivary gland cancer	5	0	1	1	1	2	4	0	0	1	0	5
Auditory cancer	8	0	0	0	4	4	7	1	0	0	7	1
Other cancer	7	5	0	0	1	1	2	0	1	4	5	2
Total	69	19	8	10	11	21	46	2	6	15	59	10

^a^ Non‐SqCC includes acinar cell carcinoma (two cases), adenoid cystic carcinoma (four cases), salivary duct carcinoma (one case), and mucinous adenocarcinoma (three cases).

### 3.2 Treatment safety and efficacy

**FIGURE 2:**
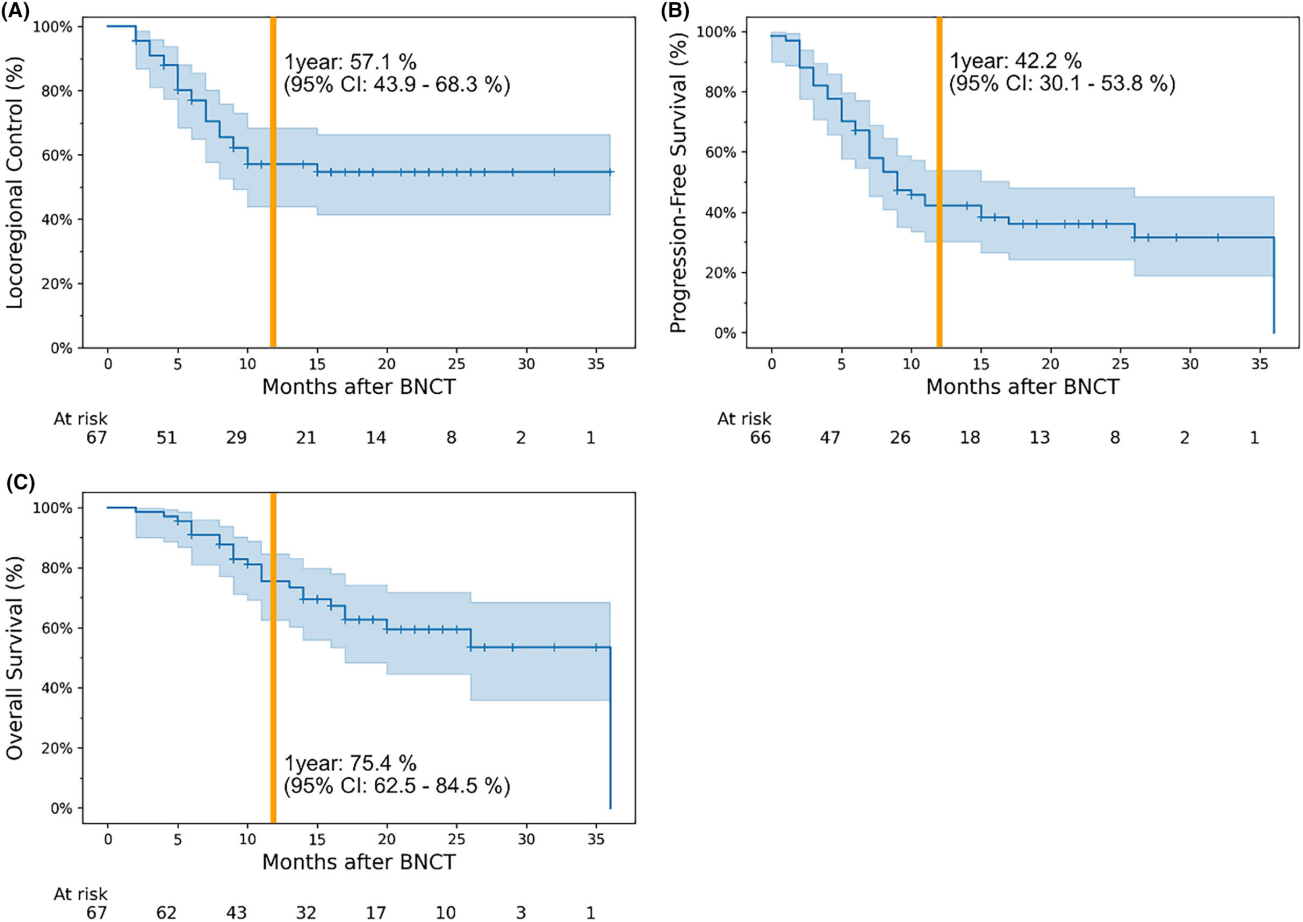
Kaplan–Meier survival curve of locoregional control (A), progression‐free survival (B), and overall survival (C). The duration of locoregional control was defined as the period from the date of irradiation to the date of disease progression at the irradiated site, and the duration of progression‐free survival was defined as the period from the date of irradiation to the date of disease progression or death. The blue area across the survival curve is the 95% confidence interval (CI). The one‐year locoregional control, progression‐free survival, and overall survival rates were 57.1% (95% CI: 43.9–68.3%), 42.2% (95% CI: 30.1–53.8%), and 75.4% (95% CI: 62.5–84.5%), respectively. The number at risk was counted at the end of each period of interest. BNCT, boron neutron capture therapy.

Complete response (CR), partial response (PR), stable disease, and progressive disease were achieved in 32, 26, 10, and 2 of the 72 total cases, respectively. The other two cases were deemed as not evaluable, due to loss to follow‐up. The objective response rate (CR + PR) was 80.5% (Table 3). Survival analysis of the 67 patients who were treated for the first time, excluding the two patients for whom there was no follow‐up data, is illustrated in Figure 2. The 1‐year locoregional control, progression‐free survival, and overall survival rates were 57.1% (95% confidence interval [CI]: 43.9%–68.3%), 42.2% (95% CI: 30.1%–53.8%), and 75.4% (95% CI: 62.5%–84.5%), respectively.

**TABLE 3:** Treatment effectiveness.

	*N*	Ratio
CR	32	44.4%
PR	26	36.1%
SD	10	13.9%
PD	2	2.8%
NE	2	2.8%

Abbreviations: CR, complete response; NE, not evaluable; PD, progressive disease; PR, partial response; SD, stable disease.

Increased serum amylase levels were observed in 50 cases (69.4% of 72 cases) as an acute Grade 3 or higher adverse event; however, all cases were asymptomatic and recovered quickly. Mucositis is inevitable when the oral cavity and pharynx are near the irradiation field, and in 5 (6.9%) cases, the patients experienced Grade 3 oral mucositis that required hospitalization. Acute kidney injury due to urinary crystallization of the boron drugs was observed in 3 (4.2%) cases; however, all of them recovered with hydration. Late adverse events due to tumor shrinkage included skin ulceration in 3 cases (4.2%), pharyngeal fistula in 2 cases (2.8%), and esophageal perforation in 1 case (1.4%). The case of esophageal perforation resulted in a Grade 5 perforation due to carotid rupture. Laryngeal necrosis was observed after irradiation in 1 case (1.4%) with hypopharyngeal cancer. In this case, although the treatment effectiveness was CR, pharyngolaryngectomy was required. Central nervous system necrosis was observed in 2 cases (2.8%), in 1 case, surgery was required (Table 4).

**TABLE 4:** Adverse event (≥ Grade 3).

	Grade3	Grade4	Grade5	All
Serum amylase increased	50			50
Mucositis oral	5			5
Dysphagia	4			4
Acute kidney injury	3			3
Skin ulceration	3			3
Pharyngeal fistula	2			2
Central nervous system necrosis	1	1		2
Pharyngeal hemorrhage	1			1
Diarrhea	1			1
Laryngeal necrosis	1			1
Malaise	1			1
Laryngeal edema^a^	1			1
Hypertension	1			1
Osteonecrosis	1			1
Esophageal perforation			1	1
Soft tissue infection	1			1
Anemia	1			1
Vagus nerve disorder	1			1
Retinopathy	1			1

^a^ The cases with tracheostomy were excluded.

### 3.3 Survival analysis

**FIGURE 3:**
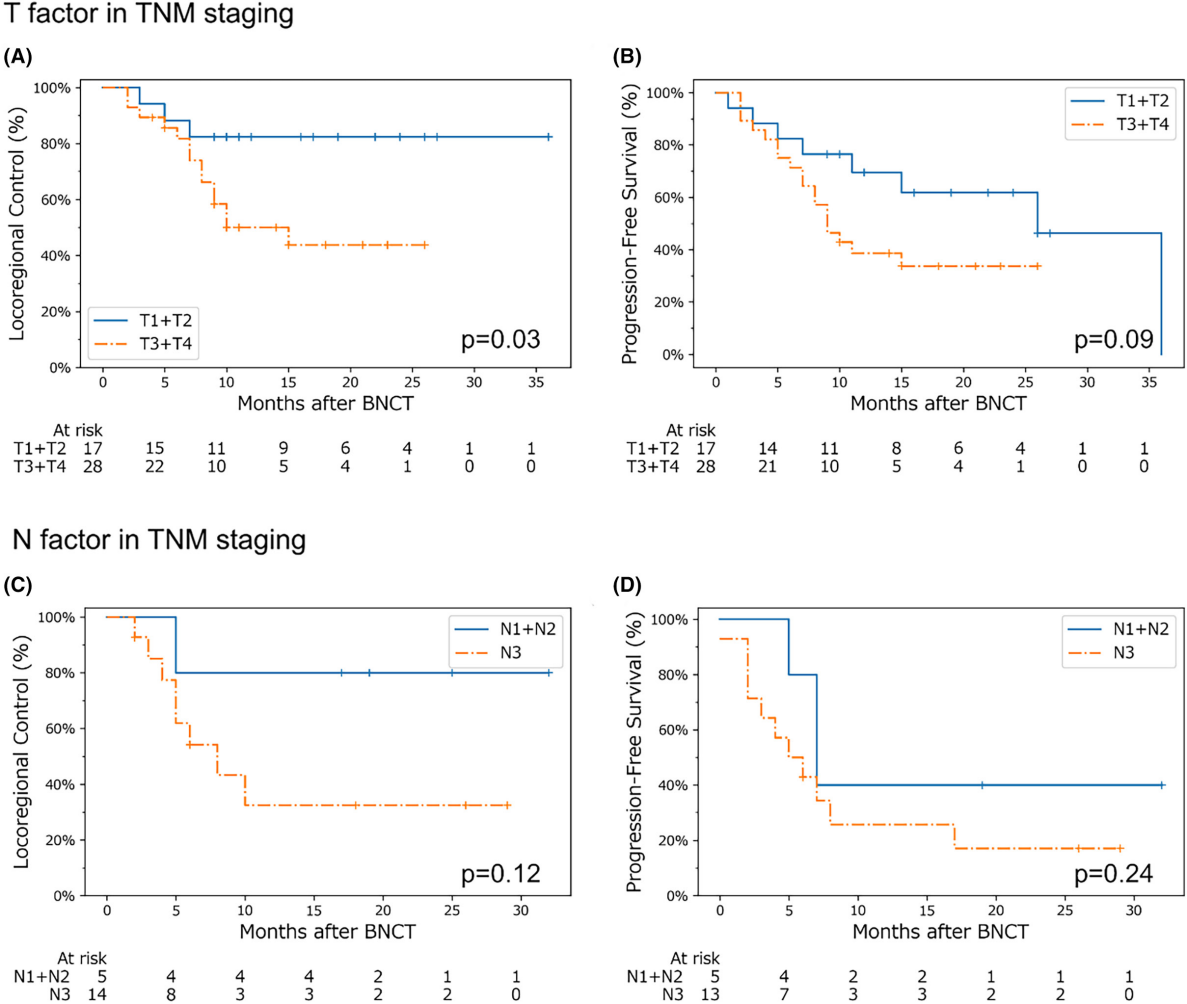
Kaplan–Meier survival curves by T factor and N factor in TNM staging. Locoregional control is shown in (A) and (C), and progression‐free survival is shown in (B) and (D). In (A) and (B), the blue and orange lines indicate T1 + T2 and T3 + T4 cases, respectively. In (C) and (D), the blue and orange lines indicate cases N1 + N2 and N3 cases, respectively. The differences between the two lines in each graph were tested using the log‐rank test, with the resultant p‐values shown in each one. The number at risk was counted at the end of each period of interest. BNCT, boron neutron capture therapy.

Our log‐rank test analysis for the patients, who were divided into two groups based on T (T1 + T2 vs. T3 + T4) and N staging (N1 + N2 vs. N3), showed that the locoregional control was significantly better in the group with early T staging (T1 + T2). The locoregional control of patients with early N stage (N1 + N2) was also favorable, although not significantly different (Figure 3).

**FIGURE 4:**
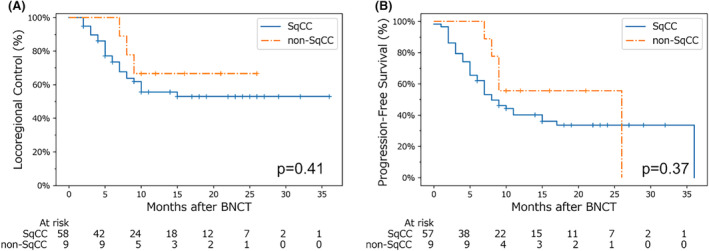
Kaplan–Meier survival curves by histological type. Locoregional control is shown in (A), and progression‐free survival is shown in (B). The blue and orange lines indicate SqCC and non‐SqCC, respectively. The difference between the two lines was tested using the log‐rank test, which yielded p‐values of 0.41 and 0.37 in (A) and (B), respectively. The number at risk was counted at the end of each period of interest. BNCT, boron neutron capture therapy; SqCC, squamous cell carcinoma.

Our analysis of locoregional control and progression‐free survival by histological type is shown in Figure 4. Prior to this analysis, a chi‐squared test confirmed there were no significant differences in terms of TNM staging between the groups, which was one of the primary factors that affected the locoregional control rate in a previous analysis (Figure 3). No significant differences were found between patients in the SqCC and non‐SqCC groups.

**FIGURE 5:**
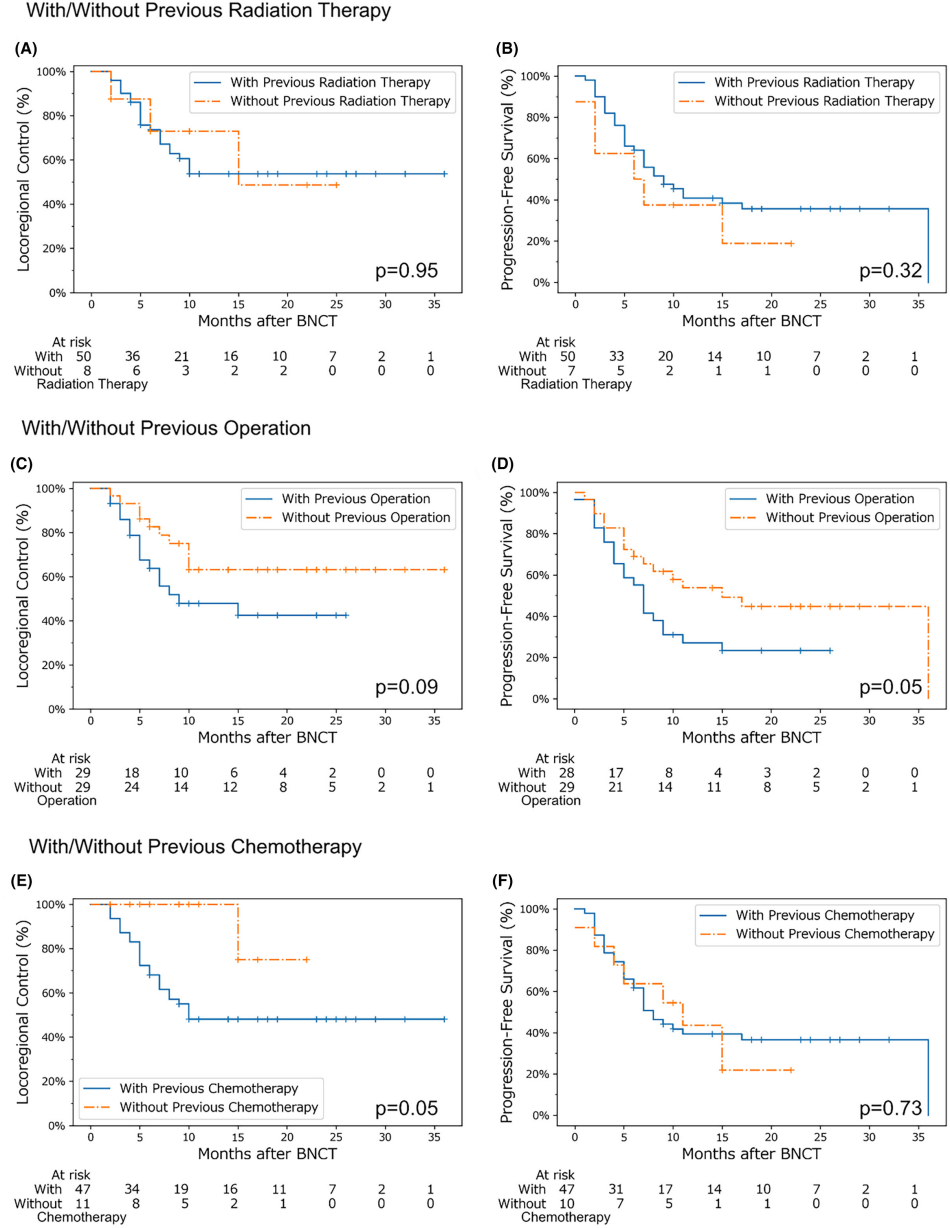
Kaplan–Meier survival curves by pretreatment in SqCC. Locoregional control is shown in (A), (C), and (E); and progression‐free survival is shown in (B), (D), and (F). In (A) and (B), the blue and orange lines indicate cases with and without previous radiation therapy, respectively. In (C) and (D), the blue and orange lines indicate cases with and without previous surgical treatments, respectively. (E) and (F), the blue and orange lines indicate cases with and without previous chemotherapy, respectively. The differences between the two lines in each graph were tested using the log‐rank test, with the resultant p‐values shown in each one. The number at risk was counted at the end of each period of interest. BNCT, boron neutron capture therapy; SqCC, squamous cell carcinoma.

Survival analysis with and without surgery, radiation therapy, and chemotherapy (including molecular‐targeted drugs) as pretreatments for BNCT was performed, focusing on SqCC cases. We confirmed beforehand that there were no significant differences in TNM staging between the groups. These results indicated that radiation therapy before BNCT did not affect locoregional control and progression‐free survival in our study cohort. However, cases treated surgically prior to BNCT tended to have worse locoregional control, and significantly lower progression‐free survival. Furthermore, locoregional control was significantly lower in patients who received chemotherapy prior to BNCT (Figure 5).

## 4 DISCUSSION

### 4.1 Safety and efficacy of BNCT

In this study, the objective response rate to BNCT was 80.5%, and the 1‐year locoregional control and progression‐free survival rates were 57.1% and 42.2%, respectively (Figure 2). In the JHN002 trial, the objective response rate was 71% and the 1‐year locoregional control rate was ~50%,^15^ suggesting that our clinical results appear to be at least equal to or better than those in previous clinical trials. Notably, the 1‐year locoregional control rate in the JHN002 study was approximately 30%, whereas it was 55.6% in the present study—particularly when limited to patients with SqCC (Figure 4). However, the 1‐year overall survival rate in the JHN002 trial was 100%, whereas it was 75.4% in this study, which may be due to differences in the patients' backgrounds and tumor histology.

Mucositis was commonly observed as an acute adverse event during this treatment; however, it was considered temporary, recoverable, and acceptable. Late adverse events in this study included skin ulceration, pharyngeal fistula, and esophageal perforation, all of which were observed in cases where the tumor involved the skin and walls of the pharynx or esophagus in a full‐layer fashion. One case, in which the carotid artery ran close to the tumor, resulted in carotid artery rupture and consequently, a Grade 5 adverse event. Therefore, caution is required when evaluating these indications. However, if the tumor bordering the carotid artery does not invade the skin or pharyngeal wall, the risk of a fatal adverse event is low and is considered a relatively acceptable risk considering the tumor status, alternative treatment options, and the patient's wishes. Other adverse events in this study included necrosis of the central nervous system and laryngeal necrosis, both of which were infrequent and considered relatively acceptable for retreatment of recurrent head and neck cancers under conditions where alternative treatment options were limited.

The median follow‐up period for this study is currently 15 months; thus, longer‐term follow‐up results are awaited with much interest. Our results indicate that BNCT may represent a new curative treatment option for recurrent or locally advanced head and neck cancers which currently have no other effective treatment options.

### 4.2 Relationship between tumor staging at the time of irradiation and treatment effectiveness

We found that better locoregional control could be expected for tumors in earlier stages at the time of irradiation (Figure 3). A previous study reported that the boron compound distribution in tumors was heterogeneous,^28^, ^29^ which may be due to tumor cell heterogeneity or other factors in the tumor environment such as the presence of hypoxic regions.^30^ Moreover, advanced cancer is generally associated with heterogeneous and uncontrolled tumor growth, resulting in heterogeneous boron compound distribution. This may represent a key factor that could reduce the therapeutic efficacy of BNCT in advanced cancers.

Currently, only a single fractionation of BNCT irradiation is approved by the Japanese health insurance system; therefore, it may be necessary to develop new irradiation methods such as fractionated irradiation to overcome this problem in the future.

### 4.3 Exploring BNCT's role among standard cancer therapies

This study examined the role of BNCT in relation to surgery and radiation therapy, which are considered standard and curative cancer treatments. Our results showed that the effectiveness of BNCT did not differ with or without previous radiation therapy, and that the adverse events were considered acceptable. Nevertheless, considering the reliability of radiation therapy (for which there is now an abundance of experience), it should be given priority over BNCT when available.

Regarding the relationship between surgery prior to BNCT and survival curve, the progression‐free survival rate decreased significantly in the group that underwent surgery. Additionally, the locoregional control rate showed a decreasing trend. One possible explanation for this could be related to hemodynamic changes due to surgery and the resulting heterogeneity of boron distribution. The effect of surgery on the general conditions of patients cannot be ignored. However, it is important to note that patients who had undergone surgery and were progressing well without recurrence were not included in this study. This demonstrates the limitation of BNCT in patients with history of surgical treatments and highlights the need for further analyses. Considering that complete surgical resection can be curative, it is reasonable to perform surgery first if a tumor is resectable.

Furthermore, the relationship between chemotherapy prior to BNCT and survival curve showed that the locoregional control rate was significantly lower in the chemotherapy group. We hypothesized that tumors that develop resistance to chemotherapy may also have different uptakes of BPA; however, further validation is required to verify this assumption. In contrast, the progression‐free survival was equivalent between the chemotherapy and chemotherapy‐naïve groups. A possible explanation for this may be that many patients in the chemotherapy‐naïve group were unable to receive chemotherapy due to poor overall health, and this affected their survival in a general sense.

Based on these points, if surgery and radiation therapy (i.e., standard curative treatments) are feasible, they should be prioritized. However, BNCT may be less effective in patients who develop resistance to chemotherapy. In addition, treatments may be less effective in advanced stage tumors. Therefore, BNCT may be more effective at an early stage when tumor recurrence or remnants are evident. Notably, chemotherapy can be administered after BNCT when considering an overall cancer treatment regimen.

### 4.4 The difference in T/B ratio and tumor CBE factor between the JHN002 trial and the present study

In the JHN002 trial, a T/B ratio of 3.5 and a CBE factor of 4.0 were used, but this study set the T/B ratio to 2.5 and the CBE factor to 3.8. Here, the T/B ratio of 2.5 and 3.5 are both hypothetical values, and the true values should be individualized for each case. The CBE factor of 3.8 and 4.0 are also hypothetical values, and the true values should be individualized for each case.^31^ This difference affects only the boron neutron dose to the tumor among the BNCT dose components. The important point here is that, in the JHN002 trial, tumor doses are only reference values and are not used to determine any clinical process. Therefore, the use of different tumor dose parameters from the JHN002 trial will not affect the clinical outcome.

The advantage of using a T/B ratio of 2.5 and a CBE factor of 3.8, which are the values we used in our previous clinical trial,^12^, ^13^ is that it provides a direct comparison to our previous clinical experience and allows us to establish eligibility criteria based on our previous experience. Conversely, the use of different parameters from the JHN002 trial may make it difficult to directly compare tumor doses to other institutions. However, there is currently no consensus on tumor dose parameters, that is, T/B ratio and CBE factor. In order to advance BNCT as radiation oncology, we believe that the parameters should be individualized rather than standardized to specific values across institutions in the future. Therefore, we have prioritized the advantage of being able to use our existing clinical experience as is and have adopted a T/B ratio of 2.5 and a CBE factor of 3.8 for this study.

### 4.5 Limitations and future challenges

This study was conducted 3 years after BNCT was approved and initiated as a health insurance‐approved treatment in Japan, and the median observation period was 15 months. Therefore, this report is still in its early stages, which represents a key limitation. It should be noted that the follow‐up procedures are not standardized as they are in clinical trials because this is real‐world data. Therefore, it would be highly beneficial to accumulate more BNCT cases and assess long‐term follow‐up data. Once more cases are collected—particularly in terms of pretreatment data—it will be possible to analyze cases separately, according to various subgroups, and exclude the effects of potential confounding factors.

In addition, as this study was conducted to clarify the place of BNCT among various standard cancer treatments in the real world, posttreatment was not standardized and was left to the discretion of each individual institution. This may represent another significant limitation of the study. However, as data accumulate, it is expected to become possible to analyze the role of BNCT as a standard cancer treatment in real‐world scenarios, including in the context of different posttreatment approaches.

## 5 CONCLUSION

Based on our 2 years of clinical experience, BNCT may be an effective alternative treatment for recurrent and locally advanced head and neck cancers, especially when no other curative treatment is available. However, this treatment modality is still in its preliminary phase. Further investigation is required to determine the actual tissue dose of BNCT and its therapeutic possibilities.

## AUTHOR CONTRIBUTIONS

**Satoshi Takeno:** Conceptualization (equal); data curation (equal); formal analysis (equal); funding acquisition (equal); investigation (equal); methodology (equal); resources (equal); software (equal); visualization (equal); writing – original draft (equal); writing – review and editing (equal). **Yuki Yoshino:** Conceptualization (equal); data curation (equal); investigation (equal); resources (equal). **Teruhito Aihara:** Conceptualization (equal); data curation (equal); investigation (equal); methodology (equal); resources (equal); supervision (equal); validation (equal). **Masaaki Higashino:** Conceptualization (equal); data curation (equal); investigation (equal); resources (equal). **Yasukazu Kanai:** Conceptualization (equal); data curation (equal); resources (equal). **Naonori Hu:** Conceptualization (equal); data curation (equal); resources (equal); writing – original draft (equal); writing – review and editing (equal). **Ryo Kakino:** Conceptualization (equal); data curation (equal); resources (equal). **Ryo Kawata:** Conceptualization (equal); data curation (equal); methodology (equal); resources (equal); supervision (equal); validation (equal). **Keiji Nihei:** Conceptualization (equal); data curation (equal); methodology (equal); resources (equal); supervision (equal); validation (equal). **Koji Ono:** Conceptualization (equal); project administration (equal); supervision (equal); validation (equal).

## FUNDING INFORMATION

This work was supported by JSPS KAKENHI Grant Number JP23K14878.

## CONFLICT OF INTEREST STATEMENT

The authors declare no conflicts of interest.

## Data Availability

Research data are stored in an institutional repository and will be shared upon request to the corresponding author.
